# How Much Does a Verbal Autopsy Based Mortality Surveillance System Cost in Rural India?

**DOI:** 10.1371/journal.pone.0126410

**Published:** 2015-05-08

**Authors:** Rohina Joshi, Deversetty Praveen, Stephen Jan, Krishnam Raju, Pallab Maulik, Vivekanand Jha, Alan D. Lopez

**Affiliations:** 1 The George Institute for Global Health, Sydney, Australia; 2 University of Sydney, Sydney, Australia; 3 CARE Foundation, Hyderabad, India; 4 The George Institute for Global Health, Hyderabad, India; 5 The George Institute for Global Health, University of Oxford, Oxford, United Kingdom; 6 University of Melbourne, Melbourne, Australia; Örebro University, SWEDEN

## Abstract

**Objective:**

This paper aims to determine the cost of establishing and sustaining a verbal-autopsy based mortality surveillance system in rural India.

**Materials and Methods:**

Deaths occurring in 45 villages (population 185,629) were documented over a 4-year period from 2003–2007 by 45 non-physician healthcare workers (NPHWs) trained in data collection using a verbal autopsy tool. Causes of death were assigned by 2 physicians for the first year and by one physician for the subsequent years. Costs were calculated for training of interviewers and physicians, data collection, verbal autopsy analysis, project management and infrastructure. Costs were divided by the number of deaths and the population covered in the year.

**Results:**

Verbal-autopsies were completed for 96.7% (5786) of all deaths (5895) recorded. The annual cost in year 1 was INR 1,133,491 (USD 24,943) and the total cost per death was INR 757 (USD 16.66). These costs included training of NPHWs and physician reviewers Rs 67,025 (USD 1474), data collection INR 248,400 (USD 5466), dual physician review for cause of death assignment INR 375,000 (USD 8252), and project management INR 341,724 (USD 7520). The average annual cost to run the system each year was INR 822,717 (USD18104) and the cost per death was INR 549 (USD 12) for the next 3 years. Costs were reduced by using single physician review and shortened re-training sessions. The annual cost of running a surveillance system was INR 900,410 (USD 19814).

**Discussion:**

This study provides detailed empirical evidence of the costs involved in running a mortality surveillance site using verbal-autopsy.

## Background

The majority of low and middle income countries (LMICs) which have the highest burden of disease lack reliable information on levels and causes of death[1]. Most deaths in these countries occur out of hospitals, and are often not registered, and thus do not form part of the vital statistics of the country. Of the 192 countries that contribute to the World Health Organisation’s database, only 23, most of which are high income economies, have good quality civil death registration data[2]. Globally, more than two-thirds of deaths occur without any medical death certification[3].

Reliable and timely cause of death information is vital to guide policy debates about the optimal distribution of scare healthcare resources, health systems planning and the evaluation of health interventions. Yet many developing countries do not have the statistical and medical capability to ensure that all deaths are correctly medically certified to yield essential vital statistics for planning. The only practical option to generate this information is the widespread and routine use of verbal autopsy methods in national civil registration systems to measure cause of death patterns in underserved areas. Verbal autopsy is an epidemiological tool which has been in use for several decades to capture cause-specific mortality, initially for childhood causes of death and more recently for maternal and adult mortality[4]. It is the most practical method of obtaining cause of death data in populations without adequate vital registration and medical certification of deaths[5,6].

Typically, verbal autopsy methods have been used in research settings and in demographic surveillance systems (DSS) [4,7,8] although there is now increasing interest to apply the method in sample registration systems (SRS)[9,10] in LMICs to better understand population level cause of death patterns [3,11,12,13]. A key potential barrier to the more widespread use of verbal autopsy methods in routine mortality surveillance systems are concerns about costs.

As verbal autopsy based surveillance systems are primarily intended for use in resource poor settings, it is important to know the costs involved in establishing and running such programs. While there are several publications that describe processes and results from verbal autopsy studies, there is no literature on the costs of the different components of verbal autopsy-based surveillance in LMIC. This paper attempts to fill this critically important policy lacunae by providing empirical evidence about the costs of establishing and running a verbal autopsy based mortality surveillance system in a rural region of India.

The mortality surveillance system was established in collaboration with a local Non-Government Organisation, The Byrraju Foundation, which sponsored a rural development initiative in Andhra Pradesh which includes a significant health care component. The Foundation aimed to ensure that the scarce resources available for health care were used to effectively and reliable cause of death data were identified as essential to the decision making process. Since reliable and current data about causes of death were not available for this population, a verbal autopsy based mortality surveillance system was established[14].

## Materials and Methods

The Andhra Pradesh Rural Health Initiative (APRHI) was initiated to design and evaluate healthcare interventions in a rural region of Andhra Pradesh, South India. The mortality surveillance system was designed to record, and assign causes to all deaths in a defined population. Approvals from the institutional ethics committees of the CARE Foundation, Hyderabad, Indian Council of Medical Research and the University of Sydney were obtained prior to initiation of the study. Written informed consent was obtained from each participant (next of kin) prior to data collection.

### The surveillance sites

The mortality surveillance system was established in 45 villages covering a population of 185,628 in the East and West Godavari Districts of Andhra Pradesh. The prospective surveillance of deaths ran for 4 years (October 2003 to September 2007). The majority of the population were agricultural and aquaculture labourers with an average monthly household income of about US$50. About 54% of the population were literate. The villages were in close proximity to each other and were connected to each other and to neighbouring towns by metalled roads.

### Implementation of the surveillance system

The surveillance system involved three key groups of personnel, each responsible for a particular aspect of the work. The project coordinator, a science graduate, supervised the day-to-day functioning of the surveillance system. His main tasks were to ensure the completeness of recording of deaths and the quality of the data collected on the verbal autopsy questionnaires. He ensured that all the non-physician healthcare workers (NPHWs) were adequately trained and provided monthly reports documenting key performance indicators relating to the project. The project coordinator was assisted by a field coordinator.

Trained NPHWs were the key personnel who carried out the day-to-day activities related to the surveillance system. Each village had one NPHW who was a village resident and with 10 years of formal schooling followed by 18 months of training in primary healthcare with a focus on maternal and child health. They collected the vital statistics, ensured provision of primary healthcare to the villagers and assisted the visiting doctor at the village health centre. The NPHWs received 5-day training in verbal autopsy data collection prior to commencement of the study with refresher training after 6 months. A manual of operations was developed specifically for the implementation of the mortality surveillance system in the local language (Telugu).

**Fig 1:**
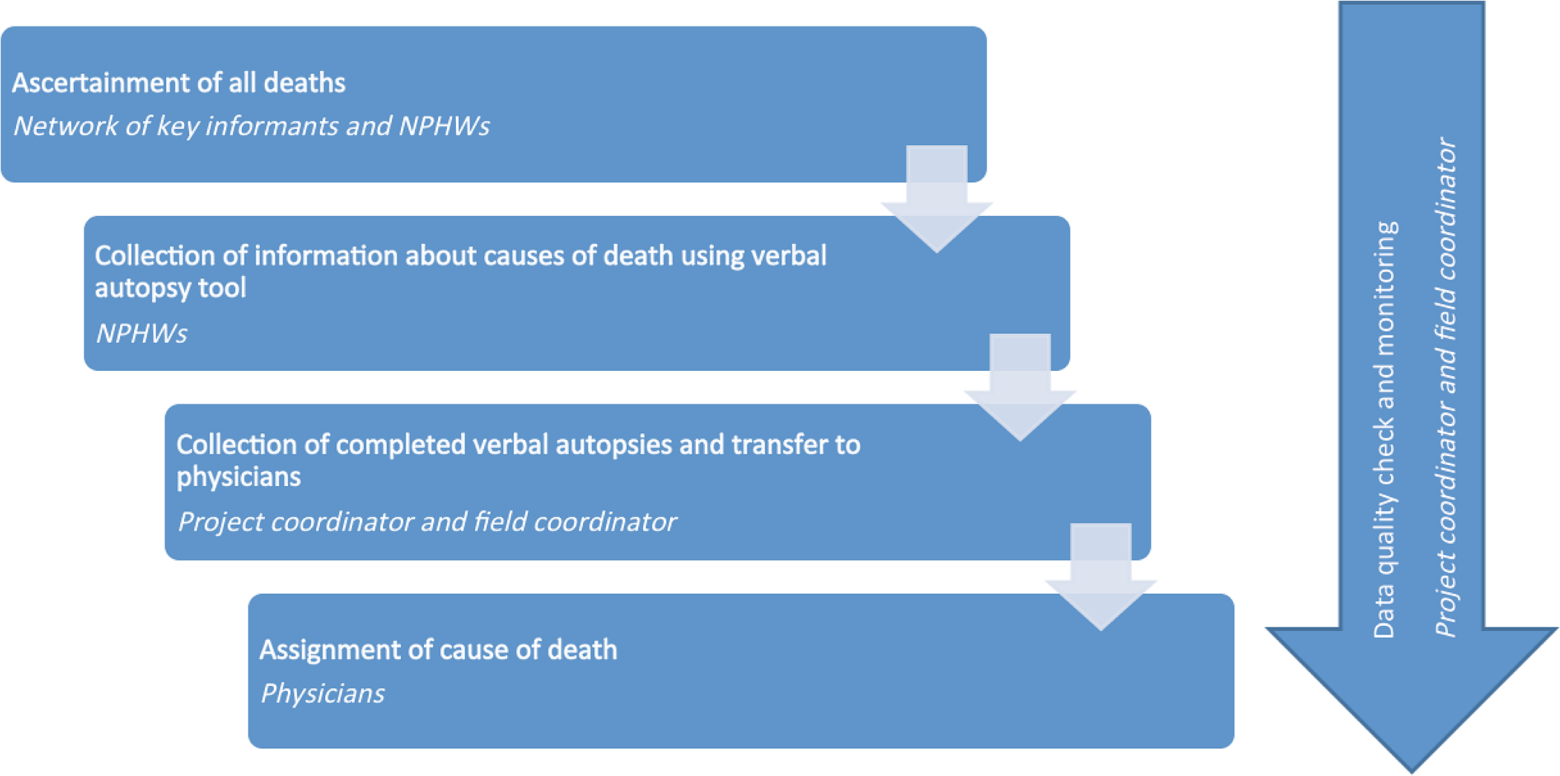
Overall design of the verbal autopsy based mortality surveillance system.

Physicians experienced in assessing verbal autopsies and diagnosing the cause of death according to the 10th version of the International Classification of Diseases were trained and engaged to assign causes of death. Most physicians had post-graduate degrees in community medicine and were fluent in the local language. Physicians were provided with training and a manual of operations developed for the cause of death assignment component of the project (Fig 1).

### Data collection

A population census was carried out in all 45 villages to establish the base population by age and sex. Trained NPHWs identified all the deaths in each village with the help of a network of key informants. This network of informants included the village headman, the ‘Panchayat’ (the local village council), priests and cremation staff, other community leaders and the government community health worker. No incentives were given to the key informants. Once a death was identified, the NPHW visited the household, identified a close relative of the deceased who was aware of the events that lead to the death, obtained an informed consent and interviewed the respondent. The NPHW visited the deceased’s household within four weeks of the date of death and conducted the verbal autopsy. The systematic inquiry about the likely cause of death was done using a verbal autopsy tool. The questionnaire had two parts, the first consisted of a series of structured questions relating to various signs and symptoms of different diseases and the second part contained an open narrative section where the respondent described the symptoms preceding death. The verbal autopsy tools used in this project were based on the questionnaires used in China[15], Tanzania[16] and the Registrar General of India’s Sample Registration System[17] with modifications made to suit local terminology (S1 Coversheet—S4 Coversheet).

Each month, the project coordinator visited the villages and collected a batch of completed verbal autopsies from the NPHW. During this visit, the project coordinator would review the number of deaths identified, check the verbal autopsy for completeness and transport it to the main office. He would then photocopy the verbal autopsy and send it to the physicians via courier.

### Cause of death assignment

In the first year of the study, questionnaires were assessed by 2 physicians trained in verbal autopsy methods, who independently arrived at a cause of death using the 10th Revision of the International Classification of Diseases (ICD-10). In the event of disagreement about the cause of death assigned, a third physician reviewed the information provided in the verbal autopsy report and decided on the diagnosis. At the end of the first year of the study (1^st^ October 2003- 30^th^ September 2004), we evaluated the impact on the observed pattern of mortality based on the verbal autopsy coding by two physicians compared to a method based on the review of a single physician. The findings showed that the physician coders provided the same assignment (at the chapter level of ICD) for 1255 (94%) of the 1329 deaths for which a verbal autopsy was done (kappa statistic 0.93 (95% confidence interval 0.92–0.94). From the second year, only one physician coder was used since there was quite close agreement about the cause of death diagnosis across the two physician reviewers[18]. For cases in which a definite cause of death could not be arrived at, a symptoms code was assigned, these were grouped and classified as ‘not elsewhere classified’. Causes of death were categorised into ICD-10 chapter headings for which death rates by age and sex were calculated. The statistical program SPSS-12 was used for analyses of causes of death.

### Cost

A top down approach was adopted to cost the surveillance system. Costs were calculated for all the processes involved in the study including data collection, diagnosis of verbal autopsies, project management and infrastructure costs to set up the surveillance system during the financial year 2004 (April 2004 to March 2005). Training costs included material costs (printing protocols, manuals, and questionnaires), costs for running a workshop (stationary, transportation, meals) and trainer fees. Costs for data collection comprised two components; salaries of NPHWs for the time spent to register deaths and complete verbal autopsies, and remuneration of physicians for coding the complete questionnaires. On average, each NPHW completed 3 verbal autopsies per month and worked on the surveillance study for approximately 1 day a week. NPHWs walked to each household to complete verbal autopsies in every village. Project staff visited villages once a month to collect and review the completed VAs. A single visit per month was used to visit villages clustered together. Project management included salaries of the project coordinator (0.5 FTE), field coordinator (0.5 FTE), photocopying of VAs, and courier costs. Start-up infrastructure costs included purchase of two desktop computers, office furniture and two mobile phones. The local study partner, Byrraju Foundation, provided office space free of cost. Research associated costs such as salaries of the researchers; international travel and accommodation were also not included since the main purpose of this paper was to describe the non-research components of establishing a VA based mortality surveillance system. Costs were calculated as a function of the average number of deaths that were registered in the year of the study, and by the population covered by the surveillance system. All costs were converted to US dollars at an exchange rate of 44.9 Indian Rupees per single US dollar (at 2003 exchange rate).

## Results and Discussion

### Start-up costs

Over the four years of the study (October 2003—September 2007), 5895 deaths were recorded of which verbal autopsies were completed for 96.7% (5786) of all deaths. The start-up costs for the study in the first year was INR 1,133,491 (USD 24,943). The total cost per death analysed was INR 757 (USD 17) and cost per capita under surveillance was INR 6 (USD 0.13). These costs included training of NPHWs and physician coders INR 67,025 (USD 1475). Training costs included photocopying of verbal autopsy tools, consent forms, participants information sheets, stationary and training manuals ($45), as well as costs for running workshops, including fees for the trainers ($550), transport and provision of meals for NPHWs ($880).

Annual costs for data collection included annual salaries of 45 NPHWs at INR 248,400 (USD 5466), dual physician review for cause of death assignment INR 375,000 (USD 8252) and project management INR 341,724 (USD 17,836).

Infrastructure costs included purchase of 2 desktop computers, a printer, 2 mobile phones, and office furniture (INR 114400, USD 264). Monthly internet connection, mobile phone fee and local transportation to visit the villages were included in the annual costs of the study (Table 1).

**Table 1:** Start-up cost of the VA based mortality surveillance (2003–2004).

Process	Cost per death	Cost per capita		Total Cost^a^		
	Indian Rupees	US dollar*	Indian Rupees	US dollar*	Indian Rupees	US dollar
***Training***	45.00	0.99	0.36	0.01	67025.00	1475.00
a) Material cost (protocol, manuals, questionnaires)						
b) Cost for running a workshop (stationary, transportation, lunch/tea)						
c) Trainer fee						
***Data collection***	166.00	3.65	1.34	0.03	248400.00	5466.40
a) Salaries of NPHWs 1day/week (45 NPHWs)						
***Cause of death assignment***	250.00	5.52	2.02	0.04	375000.00	8252.40
a) Physician fee for coding VAs (4 physicians)						
***Project management***	228.00	5.02	1.84	0.04	341742.00	7520.00
a) Salary of project coordinator 0.5FTE						
b) Salary of field coordinator 0.5 FTE						
c) Photocopying of VAs						
d) Courier costs						
***Infrastructure costs***	68.00	1.49	0.62	0.00	101342.60	2230.20
a) Computers, printers, phones						
b) Office furniture						
c) Local travel						
**Total Cost**	**757.00**	**16.67**	**6.18**	**0.12**	**1133491.60**	**24944.00**

* Conversion rate 1USD = 44.9 Indian Rupees (at 2003 exchange rate)

Note: The lifespan of the computers, printers and phones was assumed to be 4 years; hence the entire cost of the item was included in the start-up cost. The annualized cost of office furniture was estimated after adjustment for the useful life of the equipment at a discount rate of 5%.

### Cost for subsequent years

The annual cost to run the system for each of the next 3 years was INR 822,717 (USD18104), (Table 2). The cost per death was INR 550 (USD 12). Training costs for the subsequent years diminished to less than 50% of the start-up training costs. This training included an annual short re-fresher course and on the job training provided by the project coordinator. Costs for coding verbal autopsies were reduced by using single physician coding at INR 125 (USD3) per verbal autopsy coded. Project management costs were also reduced by a small amount due to the reduced costs of photocopying verbal autopsies and fewer couriers sent out due to single coding of VAs. The total cost of establishing and running the surveillance system for a population of 185,628 over 4 years was INR 3,601,643 (USD 79259) or approximately INR 4.8 (USD 0.10) per capita per year (Table 3).

**Table 2:** Cost for running the surveillance for subsequent years (2004–2007).

Process	Cost per death		Cost per capita		Total Cost^a^	
	Indian Rupees	US dollar*	Indian Rupees	US dollar*	Indian Rupees	US dollar
***Training***	21.00	0.46	0.17	0.00	31012.50	682.50
a) Material cost (protocol, manuals, questionnaires)						
b) Cost for running a workshop (stationary, transportation, lunch/tea)						
c) Trainer fee						
***Data collection***	166.00	3.65	1.34	0.03	248400.00	5466.40
**a**) Salaries of NPHWs 1day/week						
**Cause of death assignment**	125.00	2.75	1.01	0.02	187500.00	4126.20
a) Physician fee for coding VAs						
***Project management***	226.71	4.99	1.83	0.04	338862.00	7457.10
a) Salary of project coordinator 0.5FTE						
b) Salary of field coordinator 0.5 FTE						
c) Photocopying of VAs						
d) Courier costs						
***Infrastructure costs***	11.00	0.25	0.09	0.00	16942.60	372.90
a) Internet & phone connectivity						
b) Office furniture						
c) Local travel						
**Total Cost**	549.71	12.11	4.43	0.10	82271810.	18105.10

* Conversion rate 1USD = 44.9 Indian Rupees (at 2003 exchange rate)

**Table 3:** Total cost for running the surveillance system over four years.

Process	Cost per death/year		Cost per capita/year		Total Cost^a^	
	Indian Rupees	US dollar*	Indian Rupees	US dollar*	Indian Rupees	US dollar
***Training***	27.00	0.59	0.86	0.02	160062.50	3522.40
a) Material cost (protocol, manuals, questionnaires)						
b) Cost for running a workshop (stationary, transportation, lunch/tea)						
c) Trainer fee						
***Data collection***	166.00	3.65	5.35	0.12	993600.00	21865.50
a) Salaries of NPHWs 1day/week						
***Cause of death assignment***	156.25	3.44	5.05	0.11	937500.00	20630.90
a) Physician fee for coding VAs						
***Project management***	227.03	5.00	7.32	0.16	1358310.00	29891.40
a) Salary of project coordinator						
b) Salary of field coordinator						
c) Photocopying of VAs						
d) Courier costs						
***Recurrent costs***	25.25	0.56	0.89	0.00	165227.20	3636.00
a) Internet & phone connectivity						
b) Equipment						
c) Local travel						
**Total Cost**	601.53	13.24	19.47	0.41	3614700.20	79546.20

* Conversion rate 1USD = 44.9 Indian Rupees (at 2003 exchange rate)

### Data Quality

The verbal autopsy response rate remained above 90% throughout the 4 years of the study, being similarly high for all villages. The quality of physician review, measured by the proportion of unclassified deaths, was 18% in the first year; 10% in years two and three, and 16% in the final year of the study. The average time taken to complete a verbal autopsy, including obtaining respondent consent, was approximately 60 minutes.

Given the pressing health needs that exist within low income communities such as those in rural India, there are very limited resources available for information systems to provide the essential health intelligence required by planners. Recent research[19] has established that verbal autopsy methods can cost-effectively yield reliable data on causes of death in populations, but countries have been slow to implement these innovations, in part due to concerns about costs. There is no literature about the costs of establishing and maintaining a verbal autopsy system for estimating causes of death, nor on the comparative cost-effectiveness of verbal autopsy versus other data collection methods, although a framework for doing so has recently been proposed[20]. This study provides for the first time a detailed description of the costs involved in setting up and running a verbal autopsy based mortality surveillance system in rural India. There is a very sparse, almost non-existent literature on this subject, despite its obvious relevance for strategic policy discussions about health information system investments. Costs were separately estimated for establishing the system and annual running costs for each of the subsequent three years of operation of the system. In the first year of, coding of VAs by physicians was the most expensive component of costs (33%), followed by project management (30%) and salaries of NPHWs (22%).

The costs for analysing the causes of death were reduced by 50% from the second year onwards once it had been established that dual coding of VA interviews brought little additional advantage over single-coding as the physicians assigned the same code to 94% of all deaths (kappa statistics = 0.93, 95% CI 0.91–0.94)[18]. The costs of the APRHI surveillance system could have been further reduced with the use of technology. For instance, using smart phones or tablets for data collection would have increased the initial cost for purchasing the instruments, but would eliminate the recurring costs of printing, photocopying and couriers. It would also likely improve data management and data quality as inbuilt checks would reduce errors in the dataset. Similarly, using automated methods for cause of death assignment such as the tariff method[21] would further reduce costs as one of the most expensive and time consuming aspects of cause of death surveillance is physician coding. Apart from being an expensive resource, there is often limited physician availability in rural areas especially in LMIC and using them for this type of work diverts them from important clinical functions and the provision of essential health services. Using automated computer methods to identify patterns among reported symptoms has the advantage of standardizing VA diagnoses within and across countries quickly and at low-cost. Moreover, automated methods such as the tarrif method have been found to be more accurate than physician reviewers in determining both individual causes of death and population cause of death distributions[22].

Strategic decisions about investment in health information systems in LMICs are likely to be influenced by an understanding of the comparative cost-effectiveness of different data collection and analysis methods in various contexts. While this study provides important evidence about local costs of establishing and running a mortality surveillance system, a formal comparative cost-effectiveness study could not be carried out due to lack of data on the costs of running other routine mortality surveillance systems such as civil registration and periodic surveys, but also because there is no agreement on how to define the “effectiveness” of a mortality surveillance system. We have suggested how effectiveness might be crudely assessed by examining the frequency of ill-defined causes, or by consistency of findings with other, independent cause of death measurement efforts, however, this is at best roughly indicative of quality. Further research is required to reliably establish the comparative cost-effectiveness of various data collection approaches in countries as a key input into policy debates about optimal strategies for heath information system scale up.

In addition, our study has several limitations. Since the research was carried out on a population who were already participating in a rural health initiative, we did not need to include the expenses of the initial population census as the census was done prior to the initiation of the study. Infrastructure costs such as office space, electricity, water supply were excluded from the analysis since these were provided to the study by the local collaborators as in-kind contribution. Costs associated with initial meetings with the village elders and panchayat leaders (members of the village council) to establish the study were not collected, but would have been minimal as it involved a meeting in the village (usually a village elder’s house) over a cup of tea (about INR 200 per meeting, USD 4.40). The meeting involved a 30 minute discussion about the advantages of setting up the mortality surveillance and its impact on health interventions in the villages.

The findings from this study provide important evidence for policymakers and health professionals that it is possible to obtain reasonably reliable and timely evidence about causes of death in populations at relatively low-cost and adds to the extremely sparse literature available regarding costs of running a VA based surveillance system. The Butajira DSS in Ethiopia reported the ongoing cost of running a surveillance system amounted to US$0.80 per person surveyed per year in 2002[8]. Another study from India that explored the costs for running a VA based surveillance system for maternal mortality reported monthly costs of US 0.23 per birth interview, although this did not include training and infrastructure costs [7].

## Conclusions

Our study demonstrates that in this rural population in India, it is feasible to establish and run a verbal autopsy based mortality surveillance system for about USD 0.10 per capita per year. If the various component costs that we have described are likely to be similar elsewhere, then our research suggests that by judicious choice of surveillance sites that are practicable and likely representative of the national population, countries can rapidly, cheaply and reliably reduce ignorance about the leading causes of death in their populations, monitor and evaluate the impact of healthcare interventions, and assist policy makers in making evidence-based decisions. This will become even more important as national and global strategies to monitor and control the massive epidemics of non-communicable diseases in poor countries become even more urgent. Verbal autopsy based mortality surveillance is a low-cost and sustainable method to reliably monitor population health and could be made even more so through the use of established automated technologies to collect data and assign causes of death.

## Supporting Information

S1 Coversheet General Module of Verbal Autopsy. (PDF)

S2 Coversheet Cover page of Verbal Autopsy. (PDF)

S3 Coversheet Adult Verbal Autopsy. (PDF)

S4 Coversheet Neonatal and Child Verbal Autopsy. (PDF)

S1 Table Cost for establishing the Andhra Pradesh Rural Health Initiative mortality surveillance system (1st October, 2003- 30th September, 2004). (PDF)
