# Supplementary material for: How Much Does a Verbal Autopsy Based Mortality Surveillance System Cost in Rural India?
Source: PLoS One. 2015 May 8;10(5):e0126410. doi: 10.1371/journal.pone.0126410 (PMC4425407; doi:10.1371/journal.pone.0126410)
Supplement: S1 Coversheet — (PDF) [file pone.0126410.s001.pdf]

Andhra Pradesh Rural Health Initiative

Verbal Autopsy General Information Module

Patient Number: I__I__I__I__I__I__I   Patient Initials: I__I__I__I__I

BEFORE THE INTERVIEW, FILL IN THIS SECTION FROM THE MEDICAL RECORDS OF THE DECEASED

SECTION 1: BACKGROUND INFORMATION ABOUT DECEASED

1.1 |__|__| / |__|__| / |__|__|__|__| Date of birth (if known)
d d m m y y y y

1.2 ______________________ Name of deceased (if known)

1.3 ______________________ Name of mother (if deceased <12 yrs)

1.4 ______________________ Name of father (if deceased <12 yrs)

1.5 Sex of deceased

- ☐ Male
- ☐ Female

1.6 |__|__| / |__|__| / |__|__|__|__| Date of Death
d d m m y y y y

1.7 |__|__| **Years** (if one year or older) — Last known age of the deceased

|__|__| **Months** (if less than one year) — *(Record "00" days if less than one day)* *(99 = "Don't know")*

|__|__| **Days** (if less than one month)

1.8 What was the marital status of deceased

- ☐ Never married
- ☐ Married
- ☐ Separated
- ☐ Divorced
- ☐ Windowed

1.9 Last known level of education attended by the deceased.

- ☐ No education
- ☐ Primary school
- ☐ Secondary school
- ☐ Post secondary
- ☐ Not listed

1.10 |__|__| **Years** — Number of years completed
9. Don't know

BEFORE THE INTERVIEW, FILL IN THIS SECTION. THERE QUESTIONS SHOULD NOT BE ASKED TO THE RESPONDENT

SECTION 1: BACKGROUND ABOUT INTERVIEW

2.1 ________________________________ Language of Interview

2.2 |__|__|__|__|__|__|__|__|__|__|__|__|__|__|__|__|__|__|__|__|__|__|__|__|__|__|__|__|__|__| Investigator name

|____| Interviewer ID number

2.3 Date of attempted and successful interviews:

I__I__I / I__I__I / I_2_I_0_I__I__I (d d / m m / y y y y)	Date of first interview attempt
I__I__I / I__I__I / I_2_I_0_I__I__I (d d / m m / y y y y) --- (Time)	Date and time arranged for second interview attempt
I__I__I / I__I__I / I_2_I_0_I__I__I (d d / m m / y y y y) --- (Time)	Date and time arranged for third interview attempt
I__I__I / I__I__I / I_2_I_0_I__I__I (d d / m m / y y y y)	Date of interview
I__I__I / I__I__I / I_2_I_0_I__I__I (d d / m m / y y y y)	Date form checked by supervisor
I__I__I / I__I__I / I_2_I_0_I__I__I (d d / m m / y y y y)	Date entered in computer

Instructions to interviewer: Introduce yourself and explain the purpose of your visit. Ask to speak to the mother or to another adult who was the deceased's main caretaker during the illness that led to death. If this is not possible, arrange a time to revisit the household when the caretaker will be home.

2.4 Address of/directions to household

2.5 Sketch a Map if Needed

Andhra Pradesh Rural Health Initiative

Verbal Autopsy General Information Module

Patient Number: I__I__I__I__I__I__I__I Patient Initials: I__I__I__I__I

INTERVIEW BEGINS

Instructions to the interviewer: Introduce yourself and explain the purpose of your visit. Ask to speak to the mother or to another adult who was the deceased's main caretaker during the illness that lead to death. If this is not possible, arrange a time to revisit the household when the caretaker will be home (see example below)

"My name is [your name]. I am an interviewer with the Andhra Pradesh Rural Health Initiative. I have bee informed that a death has occurred in your household. I am very sorry to hear that a member of your household has passed away. Please accept my sympathies. For the purpose of improving health care, we are collecting information on all recent deaths in this area. I would like to talk to the mother of the main caretaker of [the deceased's name] and ask some questions about the events and any symptoms that [the deceased's name] had during her/his illness before death.'

SECTION 3: CONSENT

INTERVIEWER: Read the consent form to the respondent. Ask the respondent if he or she has any questions. Once any questions are answered, ask the respondent if he or she is willing to take part in the study. If so, ask them to sign the consent form.

3.1 Y N *Did respondent sign the consent form?*

→ **If yes, *proceed to 3.1.***

If answer is "No" then thank respondent for their time and end the interview.

SECTION 4: INFORMATION ABOUT RESPONDENT

4.1 ______________________________ What is your (the respondent's) name?

4.2 Interviewer: What is the sex of respondent?

Y Male

Y Female

4.3 What is your (the respondent's) relationship to the deceased? (*tick relevant box*)

- Y Mother
- Y Father
- Y Grandmother
- Y Grandfather
- Y Aunt
- Y Uncle
- Y Husband
- Y Wife
- Y Brother
- Y Sister
- Y Birth attendant, type______________
- Y Other Male, specify ______________
- Y Other Female, specify ______________

4.4 |__|__| **Years** How old are you?

4.5 What was the last level of school you attended?

- Y No education
- Y Primary school
- Y Secondary school
- Y Post secondary
- Y Not listed

|__|__| **Numbers** How many people live at this address?

4.6 |__|__| **Numbers** How many rooms are there in your home? *(None = 0)*

4.7 Does your household have a separate room for cooking?

- Y Yes
- Y No

4.8 Do you or anyone in your household have (mark all that apply)

1. Electricity?
   - Y
   - Y
2. Radio?
   - Y Yes
   - Y No
3. Television?
   - Y Yes
   - Y No
4. Refrigerator?

- Yes
- No

5. Fixed telephone line?
- Yes
- No

6. Mobile/cellular telephone?
- Yes
- No

7. Computer?
- Yes
- No

8. Car/truck?
- Yes
- No

9. Piped water inside the residence?
- Yes
- No

10. Piped water outside the residence?
- Yes
- No

11. Flush toilet inside the residence?
- Yes
- No

12. Flush toilet inside the residence?
- Yes
- No

SECTION 5: QUESTIONS ON THE DECEASED

1.11 |__|__| / |__|__| / |__|__|__|__| When was the deceased born?
d d m m y y y y

1.12 What was the sex of the deceased?
- Male
- Female

1.13 |__|__| / |__|__| / |__|__|__|__| Date of Death
d d m m y y y y

STOP.
If deceased was <12 years old, begin Neonatal/Child VA Instrument
If deceased was 12 years or older, begin Adult VA Instrument
