# Supplementary material for: How Much Does a Verbal Autopsy Based Mortality Surveillance System Cost in Rural India?
Source: PLoS One. 2015 May 8;10(5):e0126410. doi: 10.1371/journal.pone.0126410 (PMC4425407; doi:10.1371/journal.pone.0126410)
Supplement: S2 Coversheet — (PDF) [file pone.0126410.s002.pdf]

Andhra Pradesh Rural Health Initiative

Verbal Autopsy Coversheet

Patient Number: I__I__I__I__I__I__I   Patient Initials: I__I__I__I__I

SECTION 1: BACKGROUND

1.1 ____ Language of Interview

1.2 I__I__I__I__I__I__I__I__I__I__I__I__I__I__I__I__I__I__I__I__I__I__I__I__I__I__I__I__I__I I Investigator name

1.3 I____I Interviewer ID number

1.4 I__I__I / I__I__I / I_2_I_0_I__I__I (d d / m m / y y y y) Date of attempted and successful interviews:

I__I__I / I__I__I / I_2_I_0_I__I__I (d d / m m / y y y y) Date of first interview attempt

I__I__I / I__I__I / I_2_I_0_I__I__I (d d / m m / y y y y) Date and time arranged for second interview attempt

I__I__I / I__I__I / I_2_I_0_I__I__I (d d / m m / y y y y) Date and time arranged for third interview attempt

I__I__I / I__I__I / I_2_I_0_I__I__I (d d / m m / y y y y) Date of interview

I__I__I / I__I__I / I_2_I_0_I__I__I (d d / m m / y y y y) Date form checked by supervisor

I__I__I / I__I__I / I_2_I_0_I__I__I (d d / m m / y y y y) Date entered in computer

Instructions to interviewer: Introduce yourself and explain the purpose of your visit. Ask to speak to the mother or to another adult who was the deceased's main caretaker during the illness that led to death. If this is not possible, arrange a time to revisit the household when the caretaker will be home.

1.5 Address of/directions to household

1.6 Sketch a Map if Needed

SECTION 2: CONSENT

INTERVIEWER: Read the consent form to the respondent. Ask the respondent if he or she has any questions. Once any questions are answered, ask the respondent if he or she is willing to take part in the study. If so, ask them to sign the consent form.

2.1 Y N *Did respondent sign the consent form?*

→ **If yes, *proceed to 3.1.***

If answer is "No" then thank respondent for their time and end the interview.

SECTION 3: INFORMATION ABOUT RESPONDENT

3.1 ____ Name of main respondent

3.2 What is the relationship of main respondent to the deceased? (*tick relevant box*)

- Y Mother
- Y Father
- Y Grandmother
- Y Grandfather
- Y Aunt
- Y Uncle
- Y Birth attendant, type______________
- Y Other Male, specify ______________
- Y Other Female, specify ______________

3.3 |__|__| **Years** What is the age of main respondent?

3.4 |__|__| **Years** How many years of school did the main respondent complete?

3.5 Y N Were there other people present at the time of the interview?

→ **If yes,** *provide the below information for any additional respondents.*

If "No" go to 4.1

3.6 Mother
- Y N Present during illness
- Y N Present at Death

3.7 Father
- Y N Present during illness
- Y N Present at Death

3.8 Aunt
- Y N Present during illness
- Y N Present at Death

3.9 Uncle
- Y N Present during illness
- Y N Present at Death

3.10 Grandfather
- Y N Present during illness
- Y N Present at Death

3.11 Grandmother
- Y N Present during illness
- Y N Present at Death

3.12 Traditional Birth Attendant

- Y N Present during illness
- Y N Present at Death

3.13 Other Male, specify_______________

- Y N Present during illness
- Y N Present at Death

3.14 Other Female, specify_______________

- Y N Present during illness
- Y N Present at Death

3.15 |__|__| **Numbers** How many people live at this address?

3.16 |__|__| **Numbers** How many rooms are there in your home? *(None = 0)*

3.17 |__|__| **Numbers** How many chairs are there in your home? *(None = 0)*

3.18 |__|__| **Numbers** How many tables are there in your home? *(None = 0)*

3.19 |__|__| **Numbers** How many cars are there in your household? *(None = 0)*

3.20 Does your home have electricity?

- Y Yes
- Y No
- Y Refused to answer
- Y Don't know

3.21 Does anyone in your household have a bicycle?

- Y Yes
- Y No
- Y Refused to answer
- Y Don't know

3.22 Does anyone in your household have a clock?

- Y Yes
- Y No
- Y Refused to answer
- Y Don't know

3.23 Does anyone in your household have a bucket?

- Y Yes
- Y No
- Y Refused to answer
- Y Don't know

3.24 Does anyone in your household have a washing machine for clothes?

- Y Yes
- Y No
- Y Refused to answer
- Y Don't know

3.25 Does anyone in your household have a washing machine for dishes?

- [ ] Yes
- [ ] No
- [ ] Refused to answer
- [ ] Don't know

3.26 Does anyone in your household have a refrigerator?

- [ ] Yes
- [ ] No
- [ ] Refused to answer
- [ ] Don't know

3.27 Does anyone in your household have a fixed telephone line?

- [ ] Yes
- [ ] No
- [ ] Refused to answer
- [ ] Don't know

3.28 Does anyone in your household have a mobile/cellular telephone?

- [ ] Yes
- [ ] No
- [ ] Refused to answer
- [ ] Don't know

3.29 Does anyone in your household have a television?

- [ ] Yes
- [ ] No
- [ ] Refused to answer
- [ ] Don't know

3.30 Does anyone in your household have a computer?

- [ ] Yes
- [ ] No
- [ ] Refused to answer
- [ ] Don't know

SECTION 4: BACKGROUND INFORMATION ABOUT DECEASED

4.1 |__|__| / |__|__| / |__|__|__|__| Date of birth (if known)
d d m m y y y y

4.2 ____________________ Name of deceased (if >1 month)

4.3 ____________________ Name of mother (if deceased <12 yrs)

4.4 ____________________ Name of father (if deceased <12 yrs)

4.5 Sex of deceased

- [ ] Male
- [ ] Female

4.6 |__|__| / |__|__| / |__|__|__|__| Date of Death
d d m m y y y y

4.7 |__|__| **Days** Last known age of the deceased

OR

|__|__| **Years** *(Record “00” days if stillbirth)*
(99 = “Don’t know”)

4.8 What was the marital status of deceased

- ☐ Never married
- ☐ Married
- ☐ Separated
- ☐ Divorced
- ☐ Windowed

4.9 |__|__| **Years** Completed years of education of the deceased
(99 = Don’t know)

STOP.

If deceased was <12 years old, begin Neonatal/Child VA Instrument

If deceased was 12 years or older, begin Adult VA Instrument
