# Supplementary material for: How Much Does a Verbal Autopsy Based Mortality Surveillance System Cost in Rural India?
Source: PLoS One. 2015 May 8;10(5):e0126410. doi: 10.1371/journal.pone.0126410 (PMC4425407; doi:10.1371/journal.pone.0126410)
Supplement: S3 Coversheet — (PDF) [file pone.0126410.s003.pdf]

Andhra Pradesh Rural Health Initiative

Adult Verbal Autopsy

Byrraju FOUNDATION

THE GEORGE INSTITUTE *for International Health*

Patient Number: I__I__I__I__I__I__I   Patient Initials: I__I__I__I__I

SECTION 1: HISTORY OF CHRONIC CONDITIONS OF THE DECEASED

1.1 Did _______ have any of the following?

- AIDS
  - Yes
  - No
  - Refused to answer
  - Don’t know
- Asthma
  - Yes
  - No
  - Refused to answer
  - Don’t know
- Arthritis
  - Yes
  - No
  - Refused to answer
  - Don’t know
- Chronic Bronchitis
  - Yes
  - No
  - Refused to answer
  - Don’t know
- Dementia
  - Yes
  - No
  - Refused to answer
  - Don’t know
- Depression
  - Yes
  - No
  - Refused to answer
  - Don’t know
- Diabetes
  - Yes
  - No
  - Refused to answer
  - Don’t know
- Emphysema
  - Yes
  - No
  - Refused to answer
  - Don’t know

- Epilepsy
  - Y Yes
  - Y No
  - Y Refused to answer
  - Y Don't know
- Heart Disease
  - Y Yes
  - Y No
  - Y Refused to answer
  - Y Don't know
- High Blood Pressure
  - Y Yes
  - Y No
  - Y Refused to answer
  - Y Don't know
- Obesity
  - Y Yes
  - Y No
  - Y Refused to answer
  - Y Don't know
- Stroke
  - Y Yes
  - Y No
  - Y Refused to answer
  - Y Don't know
- Tuberculosis
  - Y Yes
  - Y No
  - Y Refused to answer
  - Y Don't know

SECTION 2: INJURIES/ACCIDENTS

2.1 Did ________ suffer from an injury or accident such as a…….?
Ask respondent each in sequence and mark all to which the respondent indicated "Yes."

- Y Road traffic injury
- Y Fall
- Y Drowning
- Y Poisoning
- Y Bite or sting by venomous animal
- Y Burn
- Y Violence (suicide, homicide, abuse)
- Y Other injury, Specify____________________

If the answer to all of the above was "No" go to 3.1.

2.2 Was the injury or accident self-inflected?

- Yes
- No
- Refused to answer
- Don't know

2.3 Was the injury or accident intentionally inflicted by someone else?

- Yes
- No
- Refused to answer
- Don't know

2.4 |__|__| **Hours** How long did ________ survive after the injury?

|__|__| **Days**

- Refused to answer
- Don't know

SECTION 3: SYMPTOM CHECKLIST

3.1 |__|__| **Months** For how long was ______ ill before s/he died?

|__|__| **Days**

- Refused to answer
- Don't know

3.2 Did _____ have a fever?

- Yes
- No
- Refused to answer
- Don't know

If "No" or "Refused to answer" or "Don't know" go to question 3.6.

3.3 |__|__| **Days** How many days did the fever last?

- Refused to answer
- Don't know

3.4 How severe was the fever?

- Mild
- Moderate
- Severe
- Refused to answer
- Don't know

3.5 What was the pattern of fever?

- Continuous
- On and off
- Only at night
- Refused to answer
- Don't know

3.6 Did ______ have sweating?
- Yes
- No
- Refused to answer
- Don't know

3.7 Did _____ have a rash?
- Yes
- No
- Refused to answer
- Don't know

If "No" or "Refused to answer" or "Don't know" go to question 3.10.

3.8 |__|__| **Days** How many days did ______ have the rash?
- Refused to answer
- Don't know

3.9 Where was the rash located?
- Face
- Trunk
- Extremities
- Everywhere

3.10 Did ____ have sores?
- Yes
- No
- Refused to answer
- Don't know

If "No" or "Refused to answer" or "Don't know" go to question 3.12

3.11 Did the sores have clear fluid or pus?
- Yes
- No
- Refused to answer
- Don't know

3.12 Did ______ have itching of skin?
- Yes
- No
- Refused to answer
- Don't know

3.13 Did ______ have an ulcer (pit) on the foot?
- Yes
- No
- Refused to answer
- Don't know

If "No" or "Refused to answer" or "Don't know" go to question 3.16.

3.14 Did the ulcer ooze pus?

☐ Yes
☐ No
☐ Refused to answer
☐ Don't know

If "No" or "Refused to answer" or "Don't know" go to question 3.16.

3.15 |__|__| **Days** For how many days did the ulcer ooze pus?

☐ Refused to answer
☐ Don't know

3.16 Did _______ experience "pins and needles" in their feet?

☐ Yes
☐ No
☐ Refused to answer
☐ Don't know

3.17 Did _______ have blue lips?

☐ Yes
☐ No
☐ Refused to answer
☐ Don't know

3.18 Had ______ lost weight in the three months prior to death?

☐ Yes
☐ No
☐ Refused to answer
☐ Don't know

If "No" or "Refused to answer" or "Don't know" go to question 3.20.

3.19 How substantial was the loss of weight?

☐ Slight
☐ Moderate
☐ Large
☐ Refused to answer
☐ Don't know

3.20 Did _____ look pale?

☐ Yes
☐ No
☐ Refused to answer
☐ Don't know

3.21 Did _____ have yellow discoloration of eyes?

☐ Yes
☐ No
☐ Refused to answer
☐ Don't know

If "No" or "Refused to answer" or "Don't know" go to question 3.33.

3.22 |__|__| **Months** For how long did ______ have the yellow discoloration?
|__|__| **Days**
☐ Refused to answer
☐ Don't know

3.23 Did _____ have ankle swelling?
☐ Yes
☐ No
☐ Refused to answer
☐ Don't know

If "No" or "Refused to answer" or "Don't know" go to question 3.35.

3.24 |__|__| **Months** For how long did ______ have ankle swelling?
|__|__| **Days**
☐ Refused to answer
☐ Don't know

3.25 Did _____ have puffiness of the face?
☐ Yes
☐ No
☐ Refused to answer
☐ Don't know

If "No" or "Refused to answer" or "Don't know" go to question 3.37.

3.26 |__|__| **Months** For how long did ______ have puffiness of the face?
|__|__| **Days**
☐ Refused to answer
☐ Don't know

3.27 Did _______ have general puffiness all over his/her body?
☐ Yes
☐ No
☐ Refused to answer
☐ Don't know

If "No" or "Refused to answer" or "Don't know" go to question 3.39.

3.28 |__|__| **Months** For how long did _______ have puffiness all over his/her body?
|__|__| **Days**
☐ Refused to answer
☐ Don't know

3.29 Did _____ have a lump in the neck?
☐ Yes
☐ No
☐ Refused to answer
☐ Don't know

3.30 Did _____ have a lump in the armpit?
- Yes
- No
- Refused to answer
- Don't know

3.31 Did _____ have glandular swelling in the groin?
- Yes
- No
- Refused to answer
- Don't know

3.32 Did _____ have a cough?
- Yes
- No
- Refused to answer
- Don't know

If "No" or "Refused to answer" or "Don't know" go to question 3.46.

3.33 |__|__| **Months** For how long did ______ have a cough?
|__|__| **Days**
- Refused to answer
- Don't know

3.34 Did the cough produce sputum?
- Yes
- No
- Refused to answer
- Don't know

3.35 Did _____ cough blood?
- Yes
- No
- Refused to answer
- Don't know

3.36 Did _____ have difficulty breathing?
- Yes
- No
- Refused to answer
- Don't know

If "No" or "Refused to answer" or "Don't know" go to question 3.50.

3.37 |__|__| **Months** For how long did ____ have difficulty breathing?
|__|__| **Days**
- Refused to answer
- Don't know

3.38 Was the difficulty continuous or on and off?
- ☐ Continuous
- ☐ On and off
- ☐ Refused to answer
- ☐ Don't know

3.39 In what position did the difficulty get worse?
- ☐ Lying
- ☐ Sitting
- ☐ Didn't matter
- ☐ Refused to answer
- ☐ Don't know

3.40 Did _____ have fast breathing?
- ☐ Yes
- ☐ No
- ☐ Refused to answer
- ☐ Don't know

If "No" or "Refused to answer" or "Don't know" go to question 3.52.

3.41 For how long did ______ have fast breathing?
- |__|__| **Months**
- |__|__| **Days**
- ☐ Refused to answer
- ☐ Don't know

3.42 Did _______ wheeze? *(Demonstrate)*
- ☐ Yes
- ☐ No
- ☐ Refused to answer
- ☐ Don't know

3.43 Did _____ experience pain or discomfort in the chest in the month preceding death?
- ☐ Yes
- ☐ No
- ☐ Refused to answer
- ☐ Don't know

If "No" or "Refused to answer" or "Don't know" go to question 3.57.

3.44 How long did the pain or discomfort last?
- ☐ Less than 30 minutes
- ☐ 30 minutes to 24 hours
- ☐ More than 24 hours
- ☐ Refused to answer
- ☐ Don't know

3.45 Was the pain or discomfort during physical activity?
- ☐ Yes
- ☐ No
- ☐ Refused to answer
- ☐ Don't know

3.46 Where was the pain or discomfort located? *(Read each choice in sequence.)*

- Upper or middle chest
- Lower chest
- Left arm
- Other, specify___________________
- Refused to answer
- Don't know

3.47 Did _____ have more frequent loose or liquid stools than usual?

- Yes
- No
- Refused to answer
- Don't know

If "No" or "Refused to answer" or "Don't know" go to question 3.59.

3.48 |__|__| **Days** For how long before death did _____ have loose or liquid stools?

- Refused to answer
- Don't know

3.49 Did _____ have a change in bowel habits?

- Yes
- No
- Refused to answer
- Don't know

3.50 Was there blood in the stool?

- Yes
- No
- Refused to answer
- Don't know

If "No" or "Refused to answer" or "Don't know" go to question 3.62.

3.51 Was there blood in the stool up until death?

- Yes
- No
- Refused to answer
- Don't know

3.52 Did _________ stop urinating?

- Yes
- No
- Refused to answer
- Don't know

3.53 Did _____ vomit?

- Yes
- No
- Refused to answer
- Don't know

If "No" or "Refused to answer" or "Don't know" go to question 3.67.

3.54 |__|__| **Days**   For how long before death did ______ vomit?
|__|__| **Hours**
- ☐ Refused to answer
- ☐ Don’t know

3.55 Was there blood in the vomit?
- ☐ Yes
- ☐ No
- ☐ Refused to answer
- ☐ Don’t know

3.56 Was the vomit black?
- ☐ Yes
- ☐ No
- ☐ Refused to answer
- ☐ Don’t know

3.57 Did _____ have difficulty swallowing?
- ☐ Yes
- ☐ No
- ☐ Refused to answer
- ☐ Don’t know

If “No” or “Refused to answer” or “Don’t know” go to question 3.70.

3.58 |__|__| **Months**   For how long before death did ______ have difficulty swallowing?
|__|__| **Days**
- ☐ Refused to answer
- ☐ Don’t know

3.59 Was the difficulty with swallowing with solids, liquids, or both?
- ☐ Solids
- ☐ Liquids
- ☐ Both
- ☐ Refused to answer
- ☐ Don’t know

3.60 Did ________ have pain upon swallowing?
- ☐ Yes
- ☐ No
- ☐ Refused to answer
- ☐ Don’t know

3.61 Did _____ have belly pain?
- ☐ Yes
- ☐ No
- ☐ Refused to answer
- ☐ Don’t know

If “No” or “Refused to answer” or “Don’t know” go to question 3.74.

3.62 |__|__| **Days** For how long before death did ______ have belly pain?
|__|__| **Hours**
- ☐ Refused to answer
- ☐ Don't know

3.63 Was the pain in the upper or lower belly?
- ☐ Upper belly
- ☐ Lower belly

3.64 Did _____ have a more than usual protruding belly?
- ☐ Yes
- ☐ No
- ☐ Refused to answer
- ☐ Don't know

If "No" or "Refused to answer" or "Don't know" go to question 3.77.

3.65 |__|__| **Months** For how long before death did ______ have a protruding belly?
|__|__| **Days**
- ☐ Refused to answer
- ☐ Don't know

3.66 How rapidly did _______ develop the protruding belly?
- ☐ Rapidly
- ☐ Slowly
- ☐ Refused to answer
- ☐ Don't know

3.67 Did _____ have any mass in the belly?
- ☐ Yes
- ☐ No
- ☐ Refused to answer
- ☐ Don't know

If "No" or "Refused to answer" or "Don't know" go to question 3.79.

3.68 |__|__| **Months** For how long before death did ______ have a mass in the belly?
|__|__| **Days**
- ☐ Refused to answer
- ☐ Don't know

3.69 Did _____ have headaches?
- ☐ Yes
- ☐ No
- ☐ Refused to answer
- ☐ Don't know

If "No" or "Refused to answer" or "Don't know" go to question 3.82.

3.70 |__|__| **Hours** For how long before death did ______ have headaches?
|__|__| **Days**
- ☐ Refused to answer
- ☐ Don't know

3.71 Was the onset of the headache fast or slow?
- ☐ Fast
- ☐ Slow
- ☐ Refused to answer
- ☐ Don't know

3.72 Did _____ have a stiff neck?
- ☐ Yes
- ☐ No
- ☐ Refused to answer
- ☐ Don't know

If "No" or "Refused to answer" or "Don't know" go to question 3.84.

3.73 |__|__| **Months** For how long before death did ______ have stiff neck?
|__|__| **Days**
- ☐ Refused to answer
- ☐ Don't know

3.74 Did ______ experience a period of loss of consciousness?
- ☐ Yes
- ☐ No
- ☐ Refused to answer
- ☐ Don't know

If "No" or "Refused to answer" or "Don't know" go to question 3.88.

3.75 Did the period of loss of consciousness start suddenly or slowly?
- ☐ Suddenly
- ☐ Slowly
- ☐ Refused to answer
- ☐ Don't know

3.76 |__|__| **Hours** For how long did the period of loss of consciousness last?
|__|__| **Days**
- ☐ Refused to answer
- ☐ Don't know

3.77 Did it continue until death?
- ☐ Yes
- ☐ No
- ☐ Refused to answer
- ☐ Don't know

3.78 Did _________ experience a period of confusion at any time in the three months prior to death?
- ☐ Yes
- ☐ No
- ☐ Refused to answer
- ☐ Don't know

If "No" or "Refused to answer" or "Don't know" go to question 3.91.

3.79 |__|__| **Hours**   For how long did the period of confusion last?

|__|__| **Days**

- ☐ Refused to answer
- ☐ Don't know

3.80 Did the period of confusion start suddenly or slowly?

- ☐ Suddenly
- ☐ Slowly
- ☐ Refused to answer
- ☐ Don't know

3.81 Did ________ experience memory loss at any time in the three months prior to death?

- ☐ Yes
- ☐ No
- ☐ Refused to answer
- ☐ Don't know

3.82 Did _____ have convulsions? *(Demonstrate)*

- ☐ Yes
- ☐ No
- ☐ Refused to answer
- ☐ Don't know

If "No" or "Refused to answer" or "Don't know" go to question 3.95.

3.83 |__|__| **Minutes**   For how long before death did the convulsions last?

|__|__| **Hours**

- ☐ Refused to answer
- ☐ Don't know

3.84 Did the person become unconscious immediately after the convulsions?

- ☐ Yes
- ☐ No
- ☐ Refused to answer
- ☐ Don't know

3.85 Was _______ in any way paralyzed?

- ☐ Yes
- ☐ No
- ☐ Refused to answer
- ☐ Don't know

If "No" or "Refused to answer" or "Don't know" go to 3.98.

3.86 |__|__| **Days**   For how long before death did _______ have paralysis?

|__|__| **Months**

|__|__| **Years**

- ☐ Refused to answer
- ☐ Don't know

3.87 Which were the limbs or body parts paralyzed? *Read through the list in sequence.*

☐ Right side (hand and leg)
☐ Left side (hand and leg)
☐ Lower part of the body
☐ Upper part of the body
☐ One leg only
☐ One arm only
☐ Whole body
☐ Other, specify____________________
☐ Refused to answer
☐ Don’t know

STOP.

3.88 ***If the respondent is male then go to Section 5: Alcohol and Tobacco.***

If the respondent is female then continue to Section 4: Questions for Women.

SECTION 4: QUESTIONS FOR WOMEN

4.1 Did ________ have any swelling or lump in the breast?

☐ Yes
☐ No
☐ Refused to answer
☐ Don’t know

4.2 Did ________ have any ulcers (pits) in the breast?

☐ Yes
☐ No
☐ Refused to answer
☐ Don’t know

4.3 Did ________ have a sharp pain in the belly shortly before death?

☐ Yes
☐ No
☐ Refused to answer
☐ Don’t know

4.4 Did _______ have vaginal bleeding other than her period?

☐ Yes
☐ No
☐ Refused to answer
☐ Don’t know

4.5 Was there excessive vaginal bleeding immediately prior to death?

- Y Yes
- Y No
- Y Refused to answer
- Y Don't know

4.6 Did ______ have bad smelling vaginal discharge within 6 weeks after delivery, abortion, or miscarriage?

- Y Yes
- Y No
- Y Refused to answer
- Y Don't know

4.7 Was ________ pregnant at the time of death?

- Y Yes
- Y No
- Y Refused to answer
- Y Don't know

If "No" or "Refused to answer" or "Don't know" go to question 4.12.

4.8 |__|__| **Months** For how many months was she pregnant?

- Y Refused to answer
- Y Don't know

4.9 Did she die while in labor? (*Labor is the period of time by which contractions are less than 10 minutes apart.)*

- Y Yes
- Y No
- Y Refused to answer
- Y Don't know

If "No" or "Refused to answer" or "Don't know" go to question 4.11.

4.10 |__|__| **Hours** For how long was she in labor?

- Y Refused to answer
- Y Don't know

STOP.

Go to Section 5: Alcohol and Tobacco.

4.11 Did ______ die during an abortion?

- Y Yes
- Y No
- Y Refused to answer
- Y Don't know

STOP.

Go to Section 5: Alcohol and Tobacco.

4.12 Y N Did ______ die during an abortion or within six weeks of having an abortion?

→ **If yes,** *go to question 5.1.*

- Y Refused to answer
- Y Don't know

4.13 ☐ Y ☐ N Did ________ die within six weeks of giving birth?
→ **If yes,** *go to question 5.1.*
- ☐ Refused to answer
- ☐ Don't know

4.14 At the time of death was her period late?
- ☐ Yes
- ☐ No
- ☐ Refused to answer
- ☐ Don't know

If "No" or "Refused to answer" or "Don't know" go to question 5.1.

4.15 |__|__| **Months** For how many months was her period late?
- ☐ Refused to answer
- ☐ Don't know

SECTION 5: ALCOHOL AND TOBACCO

5.1 Did _________ use tobacco?
- ☐ Yes
- ☐ No
- ☐ Refused to answer
- ☐ Don't know

If "No" or "Refused to answer" or "Don't know" go to question 5.4.

5.2 What kind of tobacco did _____ use?
- ☐ Cigarettes
- ☐ Pipe or chewing tobacco
- ☐ Refused to answer
- ☐ Don't know

If "Pipe or chewing tobacco" or "Refused to answer" or "Don't know" go to 5.4.

5.3 |__|__| **Number** How many cigarettes did _____ smoke daily?
- ☐ Refused to answer
- ☐ Don't know

5.4 Did ______ drink alcohol?
- ☐ Yes
- ☐ No
- ☐ Refused to answer
- ☐ Don't know

If "No" or "Refused to answer" or "Don't know" go to question 6.1.

5.5 Would you say the amount of alcohol _____ drank daily was…..?
- ☐ Low
- ☐ Moderate
- ☐ High
- ☐ Refused to answer
- ☐ Don't know

SECTION 6: HEALTH RECORDS

6.1 Has a health worker ever told you what caused the death of ________?

- Yes
- No
- Refused to answer
- Don’t know

If “No” or “Refused to answer” or “Don’t know” go to question 6.3.

6.2 What did the health worker say was the cause of death?

6.3 Did _____ seek care outside the home?

- Yes
- No
- Refused to answer
- Don’t know

If “No” or “Refused to answer” or “Don’t know” go to question 6.5.

6.4 *Record the name and address of any hospital, health center, or clinic where care was sought.*

6.5 Do you have any health records that belonged to ___________?

- Yes
- No
- Refused to answer
- Don’t know

If “No” or “Refused to answer” or “Don’t know” go to question 6.9.

6.6 May I see the health records?

- Yes
- No
- Refused to answer

If “No” or “Refused to answer” or “Don’t know” go to question 6.9.

6.7 |__|__| / |__|__| / |__|__|__|__| *Record the date of the last note.*

d d m m y y y y

6.8 *Transcribe the note.*

6.9 Was a death certificate issued?

- ☐ Yes
- ☐ No
- ☐ Refused to answer
- ☐ Don't know

If "No" or "Refused to answer" or "Don't know" go to question 7.1.

6.10 May I see the death certificate?

- ☐ Yes
- ☐ No
- ☐ Refused to answer

If "No" or "Refused to answer" or "Don't know" go to question 7.1.

6.11 *Record the immediate cause of death from the certificate.*

6.12 *Record the first underlying cause of death from the certificate.*

6.13 *Record the second underlying cause of death from the certificate.*

6.14 *Record the third underlying cause of death from the certificate.*

6.15 *Record the contributing cause(s) of death from the certificate.*

SECTION 7: OPEN ENDED RESPONSE AND INTERVIEWER COMMENTS/ OBSERVATIONS

7.1 INSTRUCTIONS TO INTERVIEWER: Ask the respondent: ""Thank you for the patient responses to this exhaustive set of questions. Could you please summarize in your own words the illness leading to the death of your relative?"

Write down what the respondent tells you in his/her own words. Do not prompt except for asking whether there was anything else after the respondent finishes. Keep prompting until the respondent says there was nothing else. While recording, underline any unfamiliar terms. You may also use this space to write down your comments and observations about the interview.

END OF INTERVIEW
