# Supplementary material for: How Much Does a Verbal Autopsy Based Mortality Surveillance System Cost in Rural India?
Source: PLoS One. 2015 May 8;10(5):e0126410. doi: 10.1371/journal.pone.0126410 (PMC4425407; doi:10.1371/journal.pone.0126410)
Supplement: S1 Table — (PDF) [file pone.0126410.s005.pdf]

S1_Table: Cost for establishing the Andhra Pradesh Rural Health Initiative mortality surveillance system (1st October, 2003- 30th September, 2004)

<table>
<tr><th>Process</th><th>Cost per death</th><th colspan="3">Cost per capita</th><th colspan="2">Total Cost[a]</th></tr>
<tr><td></td><td>Indian Rupees</td><td>US dollar*</td><td>Indian Rupees</td><td>US dollar*</td><td>Indian Rupees</td><td>US dollar</td></tr>
<tr><td>Training</td><td rowspan="4">45.00</td><td rowspan="4">0.99</td><td rowspan="4">0.36</td><td rowspan="4">0.01</td><td rowspan="4">67025.00</td><td rowspan="4">1475.00</td></tr>
<tr><td>a) Material cost (protocol, manuals, questionnaires)</td></tr>
<tr><td>b) Cost for running a workshop (stationary, transportation, lunch/tea)</td></tr>
<tr><td>c) Trainer fee</td></tr>
<tr><td>Data collection</td><td rowspan="2">166.00</td><td rowspan="2">3.65</td><td rowspan="2">1.34</td><td rowspan="2">0.03</td><td rowspan="2">248400.00</td><td rowspan="2">5466.40</td></tr>
<tr><td>a) Salaries of NPHWs 1day/week (45 NPHWs)</td></tr>
<tr><td>Cause of death assignment</td><td rowspan="2">250.00</td><td rowspan="2">5.52</td><td rowspan="2">2.02</td><td rowspan="2">0.04</td><td rowspan="2">375000.00</td><td rowspan="2">8252.40</td></tr>
<tr><td>a) Physician fee for coding VAs (4 physicians)</td></tr>
<tr><td>Project management</td><td rowspan="5">228.00</td><td rowspan="5">5.02</td><td rowspan="5">1.84</td><td rowspan="5">0.04</td><td rowspan="5">341742.00</td><td rowspan="5">7520.00</td></tr>
<tr><td>a) Salary of project coordinator 0.5FTE</td></tr>
<tr><td>b) Salary of field coordinator 0.5 FTE</td></tr>
<tr><td>c) Photocopying of VAs</td></tr>
<tr><td>d) Courier costs</td></tr>
<tr><td>Infrastructure costs</td><td rowspan="4">68.00</td><td rowspan="4">1.49</td><td rowspan="4">0.62</td><td rowspan="4">0.00</td><td rowspan="4">101342.60</td><td rowspan="4">2230.20</td></tr>
<tr><td>a) Computers, printers, phones</td></tr>
<tr><td>b) Office furniture</td></tr>
<tr><td>c) Local travel</td></tr>
<tr><td>Total Cost</td><td>757.00</td><td>16.67</td><td>6.18</td><td>0.12</td><td>1133491.60</td><td>24944.00</td></tr>
</table>

Notes for calculation:

Cost per capita was calculated by dividing the total cost by the population under surveillance (185,628)
Cost per death was calculated by dividing the total cost by the number of deaths that occurred in the year.
* Conversion rate 1USD = 44.9 Indian Rupees (at 2003 exchange rate)
The lifespan of the computers, printers and phones was assumed to be 4 years; hence the entire cost of the item was included in the start-up cost. The annualized cost of office furniture was estimated after adjustment for the useful life of the equipment at a discount rate of 5%.
Research costs were excluded from this analysis.

Cost for running the surveillance for subsequent years (2004-2007)

**Process**	**Cost per death**		**Cost per capita**		**Total Cost[a]**	
	Indian Rupees	US dollar*	Indian Rupees	US dollar*	Indian Rupees	US dollar
Training	21.00	0.46	0.17	0.00	31012.50	682.50
a) Material cost (protocol, manuals, questionnaires)						
b) Cost for running a workshop (stationary, transportation, lunch/tea)						
c) Trainer fee						
Data collection	166.00	3.65	1.34	0.03	248400.00	5466.40
a) Salaries of NPHWs 1day/week						
**Cause of death assignment**	125.00	2.75	1.01	0.02	187500.00	4126.20
a) Physician fee for coding VAs						
Project management	226.71	4.99	1.83	0.04	338862.00	7457.10
a) Salary of project coordinator 0.5FTE						
b) Salary of field coordinator 0.5 FTE						
c) Photocopying of VAs						
d) Courier costs						
Infrastructure costs	11.00	0.25	0.09	0.00	16942.60	372.90
a) Internet & phone connectivity						
b) Office furniture						
c) Local travel						
**Total Cost**	549.71	12.11	4.43	0.10	82271810.	18105.10

* Conversion rate 1USD = 44.9 Indian Rupees (at 2003 exchange rate)

Total cost for running the surveillance system over four years

Process	Cost per death/year		Cost per capita/year		Total Cost[a]	
	Indian Rupees	US dollar*	Indian Rupees	US dollar*	Indian Rupees	US dollar
Training	27.00	0.59	0.86	0.02	160062.50	3522.40
a) Material cost (protocol, manuals, questionnaires)						
b) Cost for running a workshop (stationary, transportation, lunch/tea)						
c) Trainer fee						
Data collection	166.00	3.65	5.35	0.12	993600.00	21865.50
a) Salaries of NPHWs 1day/week						
Cause of death assignment	156.25	3.44	5.05	0.11	937500.00	20630.90
a) Physician fee for coding VAs						
Project management	227.03	5.00	7.32	0.16	1358310.00	29891.40
a) Salary of project coordinator						
b) Salary of field coordinator						
c) Photocopying of VAs						
d) Courier costs						
Recurrent costs	25.25	0.56	0.89	0.00	165227.20	3636.00
a) Internet & phone connectivity						
b) Equipment						
c) Local travel						
**Total Cost**	601.53	13.24	19.47	0.41	3614700.20	79546.20

* Conversion rate 1USD = 44.9 Indian Rupees (at 2003 exchange rate)
